# Estimating the level and determinants of catastrophic expenditure related to hypertension management in the Greater Accra Region of Ghana

**DOI:** 10.1080/16549716.2025.2602116

**Published:** 2025-12-18

**Authors:** Kofi Aduo-Adjei, James Akazili, Øystein Ariansen Haaland, Lumbwe Chola

**Affiliations:** a Bergen Centre for Ethics and Priority Setting (BCEPS), Department of Global Public Health and Primary Care, University of Bergen, Bergen, Norway; b School of Public Health, C.K. Tedam University of Technology and Applied Sciences (CKTUTAS), Navrongo, Ghana; c Department of Health Management and Health Economics (HELED), Institute of Health and Society, University of Oslo, Oslo, Norway

**Keywords:** hypertension, catastrophic health expenditures, survey design, Ghana, financial risk protection

## Abstract

**Background:**

Since the establishment of national health insurance in Ghana, the government’s health policy has focused on reducing out-of-pocket expenditure (OOP) to ensure that people do not become poorer while accessing healthcare services. However, studies show that OOP expenditure remains high, and a substantial portion of the population experiences catastrophic health expenditure (CHE).

**Objective(s):**

We estimated the level and determinants of catastrophic health expenditures for hypertension management among adults in the Greater Accra Region of Ghana.

**Methods:**

We conducted a cross-sectional CHE survey at two randomly selected district primary health facilities in the Greater Accra Region. Data collection took place between December 2023 and February 2024. A questionnaire was used to collect data on direct and indirect medical expenditures related to hypertension care from 382 participants. CHE was defined at thresholds of 10% and 40% of the household’s capacity for direct and indirect medical expenditures, respectively. Multivariate logistic regression was used to assess the determinants of CHE.

**Results:**

A total of 382 patients were included in the analysis; the majority were females (76%), and Christians (95%). Based on the 10% and 40% thresholds of household income capacity to pay, 65% and 58% of the total sample experienced CHE, respectively. The regression model revealed that being widowed (OR = 0.32, 95% CI: 0.09–1.10, *p = 0.07)*, unemployed (OR = 0.26, 95% CI: 0.08–0.09, *p = 0.03*), and having a large household size (OR = 1.32, 95% CI: 0.95–1.84, *p = 0.09)* correlate with CHE for hypertension management and care.

**Conclusion:**

The study demonstrates that hypertension care imposes a considerable financial burden on households.

## Paper Context

**Main findings:**

Catastrophic health expenditure for hypertension care in Ghana is driven by a complex interplay of factors spanning socioeconomic conditions, demographic characteristics, and health systems dynamics, which are unevenly distributed across population subgroups in the Greater Accra Region.

**Added knowledge:**

This empirical paper advances the substantive evidence on the economic burden of hypertension in the Greater Accra Region by employing a survey-based approach, thereby addressing a critical gap in the literature and providing insights that are of direct relevance for health policy and financial risk protection strategies.

**Global health impact for policy and action:**

This paper identifies the population groups that are at risk of catastrophic health expenditures due to hypertension care and calls for the design of a benefit package targeted at the most vulnerable households to enhance financial risk protection and reduce health inequities in Ghana.

## Background

Globally, hypertension is a major threat to public health [1]. According to the World Health Organization (WHO), about 1.3 billion individuals aged 30–79 were estimated to have hypertension in 2019 [1,2]. Only about one in five manage and control their blood pressure effectively [1], and about 11 million deaths annually are attributed to hypertensive disorders [3]. About two thirds of people with hypertension live in low- and middle-income countries (LMICs), with prevalence being highest in the WHO African region, at about 27% [1]. The Lancet Non-Communicable Diseases and Injuries Poverty Commission Report [4] indicates that high rates of hypertension are notably prevalent in Sub-Saharan Africa (SSA) – a trend that was previously more common in high-income settings [4–8]. Several factors contribute to the high prevalence of hypertension in SSA, including demographic changes, sedentary lifestyles, and behavioural, genetic, and biological factors [9]. The multifaceted nature of hypertension in SSA must be addressed by comprehensive public health strategies [9–12].

In Ghana, the prevalence of hypertension in the general population is estimated at 34.1% [10]. Its burden is predominant among women, 60% of whom are affected [13]. With regard to the urban – rural divide, hypertension affects 9% of the rural population and 16% of the urban population in Ghana [14]. The annual incidence of hypertension in health facilities in Ghana has surged dramatically, increasing more than elevenfold, from 60,000 cases in 1990 to 700,000 in 2010 [15]. In 2021, approximately 30,000 deaths were attributed to high systolic blood pressure [3]. Antihypertensive medication, which is essential for managing hypertension, is expensive, and long-term hypertension management can lead to high catastrophic health expenditure (CHE) for many households [16]. Health insurance coverage is limited in many LMICs. For instance, the Ghanaian National Health Insurance Scheme (NHIS) was established in 2003 and is financed through a 2.5% levy on goods and services, as well as a 2.5% deduction from formal sector workers’ contributions [17]. The scheme aims to improve financial access to healthcare, including coverage for hypertension services in Ghana. Studies have demonstrated the positive impact of NHIS in reducing out-of-pocket (OOP) payments and mitigating the risk of CHE, particularly among lower-income and vulnerable populations [18–20]. This evidence found that insured households were significantly less likely to incur catastrophic and impoverishment expenditures. Nonetheless, most people living with hypertension make OOP payments for medication [21]. Such inadequate financial protection increases households’ economic burdens.

Medication, consultation, and diagnostics are important drivers of hypertension-related OOP costs [22], but transportation and food costs related to treatment further contribute to high expenditures [23]. Additionally, hypertension can lead to reduced productivity due to illness, fatigue, frequent medical visits, hospitalization, and absenteeism from work. This affects the earnings of individuals and reduces the productivity of the general population. Several studies estimate that CHE in Ghana is mostly related to a lack of uptake of national health insurance [24–28]. One study [24] estimated that 11% of Ghanaian households spend more than 5% of their total household expenditure on annual OOP payments. This study further showed that illness shocks exerted a catastrophic economic burden, not only through medical expenditures but also through loss of productivity. Another study [25] indicated a greater concentration of CHE among the poorest households and substantial inequalities between the poorest and richest households. Evidence of CHE related to hypertension in Ghana is limited. This study generates evidence to inform health policies in Ghana, particularly regarding the need for financial risk protection, which is a key goal of Ghana’s Universal Health Coverage strategy. Our aim is to estimate the level of CHE among hypertensives in Ghana and to identify the associated determinants.

## Methods

### Study population and study design

**Figure 1.**
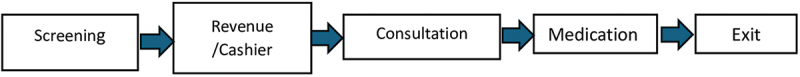
Chart depicting patient pathway.

We used an analytical cross-sectional survey design to gather data on CHE related to hypertension management in Ghana. This study was conducted in the Greater Accra Region (GAR) at Weija-Gbawe Municipal Hospital (WGMH) and Shai Osoduko District Hospital (SODH). These two facilities were purposively selected because they are integral to primary healthcare, promoting both curative and preventive care, as well as overall population health within their respective districts or municipalities. Both WGMH and SODH operate outpatient hypertension clinics that are easily accessible to the public. Additionally, their service coverage is enhanced through outreach programmes at the district level. WGMH and SODH are district and municipal health facilities with bed capacities of 100 and 91, respectively [29]. Hypertension clinics are held on Mondays and Wednesdays at WGMH, whilst patients use hypertension services on Tuesdays and Thursdays at SODH. In both facilities, nurses at the triage station examine the hypertensive patients to measure and record their vital signs. Figure 1 shows the routine care pathway for patients utilizing the hypertension clinic at SODH in the GAR, which begins at reception, followed by screening, revenue/cashier, consultation, medication, and exit from the facility. Neither facility offers on-site laboratory services, and patients typically need to seek private services for laboratory investigations.

Our survey instrument was based on the Ghana Living Standards Survey (GLSS), which includes questions on health expenditure to understand household well-being, costs associated with illness, and preventive care. It involved a review of hospital-based tools that are used to measure CHE in Sub-Saharan Africa, including facility-based surveys and the health account production tool (HAPT) [24–27]. This review informed our selection of items and questions for estimating both direct medical expenses (such as antihypertensive medications, laboratory tests, and other pharmaceutical products) and non-medical direct expenditures (such as transportation costs). The data were collected between December 2023 and February 2024. The survey gathered information on hypertension-related expenditure, management, and care practices among patients as well as participants’ sociodemographic and socioeconomic characteristics using a structured questionnaire. The questionnaire was administered face-to-face by eight experienced research assistants, who were recruited and trained in accordance with the study protocol and data collection procedures. To enhance instrument validity and reliability, the questionnaire was pilot-tested with ten patients at a health facility, and their feedback was used to amend the survey. Expenditure and income data were collected in Ghana cedis (GHS). We adopted the Central Bank of Ghana’s day-weighted average exchange rate during June 2024, which was 14.43 Ghana cedis per US dollar. This rate was used because it represents the official and most reliable market rate for that period. Using standardized and authoritative sources ensure consistency, transparency, and comparability [30].

The study sample consisted of 382 participants; this was calculated using a sample size estimation formula, based on the assumption of a *p* = 50% prevalence of hypertension [31]. This assumption was used because no reliable prior estimate of the outcome’s prevalence among clinic attendees was available, and *p* = 50% yielded the required sample size. A critical value of z = 1.96 was used, corresponding to a 95% confidence interval. The margin of error was set at 0.05 ($n=z^{2}\times p\times \left(1-p\right)/d^{2}$). The inclusion criteria for this study included participants aged 45 years and above who attended hypertension clinics at WGMH and SODH. Existing evidence suggests that the prevalence of hypertension rises sharply after the age of 40 due to age-related physiological changes [32,33]. Targeting this age group allows for a more accurate assessment of the economic burden of hypertension among those who are most at risk and most likely to experience its long-term consequences. Research assistants conducted interviews in the waiting area before patients consulted the doctor. We used a systematic approach, starting from a randomly chosen point within the hypertensive population. The sampling interval was calculated as 10 (5000/500), and every 10^th^ individual was selected until the target sample size of 382 was reached. Although the initial target sample size was 500, the study achieved 382 respondents due to non-response and practical field constraints. Systematic random sampling using an interval of 10 ensured that every individual in the population had an equal and known probability of selection, thereby reducing selection bias and improving representativeness. Of these, 192 participants were selected from SODH and 190 from WGMH.

### Data analysis

This study adopted a cost-of-illness approach from the patients’ perspective (economic perspective) to evaluate the costs incurred by hypertension patients in the Greater Accra Region of Ghana [34]. In this context, CHE includes payments made directly by patients with hypertension at the selected hospitals. We included transportation costs as they constitute a substantial portion of direct health costs [35]. In our estimation of the costs per hospital visit, we adopted two strategies.

- Limiting the analysis to only direct medical health costs.
- Combining direct medical health costs with indirect medical costs (such as transportation).

We asked the participants to report their expenses related to accessing healthcare, including transportation, food, and other costs. Transportation cost was then doubled to estimate the cost of a round trip. Participants who used private cars were asked to report the cost of fuel used when travelling from their residences to the hospital, which was also doubled to account for return journeys.

### Variable classification

The variables used in this study were defined as follows: Household (**H**) – a household was classified as a group of individuals who shared financial responsibilities; Out-of-pocket health expenditure (**OOPHE**) – the total expenditure for hypertension care, covering all services including consultation fees and medication; Total household consumption expenditure (**THE**) – comprising total expenditures by households for meeting their daily needs; and household food expenditure (**HFE**) – the total amount spent on foodstuffs, with data gathered specifically for one month [34].

### Estimation of catastrophic health expenditure

The WHO defines CHE as an economic burden that households face due to out-of-pocket health expenses. Thus, CHE occurs when a household’s healthcare spending exceeds a certain proportion of its income or capacity to pay. In this study, we adopted the 10% threshold of total household expenditure to identify households experiencing CHE, which is consistent with the WHO’s recommendations and previous studies in similar settings [34–38]. To estimate CHE, we used the WHO’s standard approach, which has been criticized for its measurement of subsistence expenditure and the relevance of the initial estimate of the equivalence scale. Recently, some authors have argued that the threshold should be based on income distribution to ensure fair and ethical measures of CHE [34]. In our study, however, the WHO’s method was deemed appropriate as it may not have been feasible to measure total household income. This method uses non-food expenditure as a reference scale and considers low-income households that may already be spending a significant portion of their budget on essential food items. It focuses on the remaining income that can be allocated to other needs, which makes it a more accurate measure of financial burden. Again, this method is widely accepted and standardized in health economics, which allows for consistency in measuring and comparing CHE across different studies and countries. Although our model explains only a modest proportion of variance, the results still offer valuable insights into the association between independent variables and catastrophic health expenditure. The seven steps of estimating CHE based on the WHO’s approach are described below.

### Step 1. Computation of food expenditure share (FES)

Food expenditure share (FES) was calculated and expressed as the ratio of household food expenditure (HFE) to total household expenditure (THE):

$$\mathrm{FES}=\frac{\mathrm{HFE}}{\mathrm{THE}}$$

### Step 2. Determination of household equivalent size (HES)

Household equivalent size (HES) was estimated from household size (hh_size_) as follows:

$$\mathrm{HES}=hh_{\mathrm{size}}^{\beta }$$

where the scale factor, β, is set to 0.56. This value was derived from a regression equation based on data from 59 countries [26]. HES allows researchers to make fair comparisons across households. Without this adjustment, larger households (household sizes with seven or more members) might appear worse off simply because they have more members, even if their per-capita resources are adequate. HES normalizes these differences, making it easier to compare living standards and incomes across households of various sizes and ensuring sensitivity in our analysis.

### Step 3. Estimation of equivalent food size (EFS)

Equivalent food size (EFS) was determined by denoting the ratio of household food expenditure (HFE) to household equivalent size (HES):

$$\mathrm{EFS}=\frac{\mathrm{HFE}}{\mathrm{HES}}$$

### Step 4. Computing subsistence expenditures (SE)

The poverty line (PL) was computed by averaging the food expenditures of households in the 45^th^ and 55^th^ percentiles of the food expenditure share (FES). The choice of these two points around the median provides a balanced measure of expenditure patterns near the centre of the distribution. By focusing on adjacent percentiles, we capture subtle variations in the household financial burden that may not be evident when considering only the median or quartiles. This approach enables greater sensitivity in detecting differences between households that are slightly below and above the midpoint. Next, we calculated subsistence expenditures (SE) as follows:

SE=PL×HES

The concept of subsistence expenditures refers to the household costs that cover their basic and essential demands, such as food, shelter, clothing, and other necessities required for survival [34]. Subsistence expenditure is adopted in a household’s capacity to pay for healthcare. This process is achieved by subtracting the amount required for basic needs from the household’s income or consumption. The remaining amount is estimated based on the household’s capacity to pay.

### Step 5. Capacity to pay (CTP)

We estimated a household’s capacity to pay (CTP) by calculating the difference between total household consumption expenditure (THE) and subsistence expenditure (SE):

$$\mathrm{CTP}=\left\{\begin{array}{c} \mathrm{THE}-\mathrm{SE}\;\mathrm{if}\;\mathrm{HFE}\;\geq \;\mathrm{SE}\\ \mathrm{THE}-\mathrm{HFE}\;\mathrm{if}\;\mathrm{HFE}\;<\;\mathrm{SE} \end{array}\right.$$

### Step 6. Catastrophic health expenditure (CHE)

Health expenditures were catastrophic when OOP health expenditure (OOPHE) exceeded a predetermined threshold, such as 10% of CTP. The 10% threshold of capacity to pay is a widely used benchmark in measuring CHE. It represents the proportion of a household’s total income or consumption expenditure that, if spent on healthcare, is considered to place a significant economic burden on the household. When out-of-pocket health payments exceed 10% of a household’s capacity to pay, it is assumed that the household may have to forgo other needs and experience financial stress [37]. In this study, applying the 10% threshold helps to quantify the extent to which hypertension-related healthcare costs become catastrophic relative to household resources. This was represented as a dummy variable, where a value of 1 was assigned if the ratio of OOPHE to CTP was greater than or equal to 10%, and 0 otherwise:

$$\mathrm{CHE}=\left\{\begin{array}{c} 1\;\mathrm{if}\;\frac{\mathrm{OOPH}}{\mathrm{CTP}}\;\geq 10\%\\ 0\;\mathrm{if}\;\frac{\mathrm{OOPH}}{\mathrm{CTP}}\;<\;10\% \end{array}\right.$$

### Step 7. Determination of household poverty status (HPS)

Household poverty status (HPS) was assessed based on the relationship between total household expenditure and subsistence expenditure. A household was classified as poor if its total household expenditure was less than its subsistence expenditure; otherwise, it was classified as non-poor:

$$\mathrm{HPS}=\left\{\begin{array}{c} 1\;\mathrm{if}\;\mathrm{THE}\;<\;\mathrm{SE}\\ 0\;\mathrm{if}\;\mathrm{THE}\geq \mathrm{SE} \end{array}\right.$$

### Determinants of catastrophic health expenditure in Ghana

We fitted a multivariate logistic regression model to identify socio-economic and sociodemographic determinants of CHE. The outcome variable was CHE (Step 6), dummy-coded for 10% threshold: direct health costs divided by the capacity to pay when equal to or greater than 10% threshold. In our model, the first outcome/dependent variable was coded as direct medical cost, and the second outcome was total health expenditure, including direct non-medical costs. The explanatory/independent variables were age, sex (Male, Female), household size, marital status (Married, Living Together, Divorced, Separated, Widowed, Never Married/Single), education (Primary, Secondary, Tertiary), employment (Employed, Unemployed), residence (Ga East Municipal Assembly, Ga South Municipal Assembly, Other), alcohol intake (Yes, No), and physical activity. We selected these variables based on the existing literature. We conducted the analysis using descriptive and multivariate statistics. Statistical computations were performed using STATA Version 18.

## Results

### Descriptive results

Table 1 shows the socioeconomic and sociodemographic characteristics of the study sample, drawn from the Greater Accra Region. As can be seen, the proportion of individuals with secondary or tertiary education was 75.5%. Labour force participation was low in the sample population, with 61.9% being unemployed. The unemployed population typically has low or no income, which makes it difficult for them to afford hypertension management medications and services. Thus, many patients are likely to delay seeking healthcare or forgo health expenditure related to hypertension management, which leads to worse health outcomes. In addition, the unemployed population in this study included both younger adults seeking work and older individuals who were not actively engaged in the labour force. The unemployed were predominantly female, which reflects the national labour market trends in which women experience higher unemployment rates. Moreover, most respondents identified as married and Christian, reflecting the broader demographic composition of the study area. Preliminary descriptive analyses indicated that several variables, including income, health expenditure, and age, were not normally distributed. To account for this, non-parametric tests were used where appropriate, and measures of central tendency (median and 25th percentiles) were reported for skewed variables. This approach ensures robust results despite deviations from normality in the underlying data.

**Table 1.** Socioeconomic and sociodemographic characteristics of study respondents.

Characteristics	Frequency	Percentage
**Gender**		
Male	89	23.3
Female	293	76.7
**Marital status**		
Married	184	48.8
Living together	20	5.3
Divorced	55	14.6
Widowed	103	27.3
Never married/Single	15	3.9
**Education**		
Primary	91	24.5
Secondary	226	60.7
Tertiary	55	14.8
**Employment status**		
Employed	138	38.0
Unemployed	225	61.9
**Residence**		
Ga East Municipal Assembly	189	49.5
Ga South Municipal Assembly	143	37.4
Other	50	13.1
**Ethnic Status**		
Akan	132	35.1
Ga-Dangme	122	32.4
Ewe	74	19.7
Guan	12	3.2
Mole-Dagbani	27	7.2
Grussi	6	1.6
Gruma	2	0.5
Mande	1	0.3
**Religion**		
Christian	359	94.9
Islam	17	4.5
Traditional/Spiritualist	1	0.3
No Religion	2	0.5
Other	3	0.8

### Cost of illness and catastrophic household and individual health expenditure

Table 2 shows that, on average, households expended 1458 Ghana cedis monthly (101 USD), with food costs averaging 803 Ghana cedis (56 USD). Subsistence mean expenditure was 1733 Ghana cedis (120 USD), indicating considerable financial strain. This reveals that many households in our study population were spending less than the minimum required to meet their basic needs, including essentials such as food, housing, clothing, and utilities. This situation suggests substantial deprivation. Thus, households may rely on borrowing, loans, and accumulating debts, which can worsen their financial situation and lead to further vulnerability. The mean capacity to pay was 74 Ghana cedis (5 USD), with negative values suggesting inability to meet subsistence needs. Direct medical costs (medication, laboratory, and consultation) per hospital visit amounted to 375 Ghana cedis (26 USD), and including transportation elevated this to 453 Ghana cedis (31 USD). Direct non-medical costs (transportation, food, and other health costs) per visit, such as transportation, averaged 40 Ghana cedis (2.7 USD), and food costs averaged 14 Ghana cedis (1 USD). The total cost of time lost per patient, including time off work, time visiting hospitals, and waiting time during visits, was estimated at 21.15 Ghana cedis (1.46 USD). Among patients who had jobs (i.e. 39%), the mean time they took off work to seek care for hypertension was 5.3 hours.

**Table 2.** Individual and household expenditure per month (*N* = 382).

Expenditure	Mean (USD)	Standard deviation	Median (USD)	Minimum	Maximum	p25
**Household costs per month**						
Total household expenditure (THE)	1458.3(101)	1040.3	1500(103)	200	14,000	800
Household food expenditure (HFE)	803.2(56)	484.2	800(55)	100	3000	500
Subsistence expenditure (SE)	1733.2(120)	353.9	2012.7(139)	710.8	3238.4	1544
Capacity to pay (CTP)	74.5 (5)	535.8	600(42)	−1044.8	4000	−100
**Direct medical cost per patient per hospital visit**						
Healthcare cost	375.4(26)	375.6	258(18)	0	3420	150
Healthcare cost (including transport)	453.4(31)	553.8	298(21)	25	6750	180
**Direct non-medical cost per patient per hospital**						
Transport cost	39.6(2.7)	107.2	20(1.3)	0	1600	10
Food cost	14.9(1)	51.1		0	920	0
**Time lost per patient (hours)**						
Time taken off work	5.3	2.8	10	1	30	4
Time taken visiting hospital	0.7	1.1	5	0.1	15	0.2
Time waiting	3.2	2.7	3	0	45	2

Mean and median expenditure is expressed in Ghana cedis, with the dollar equivalent in brackets. In this table, p25 represents the first quartile, indicating the value below which 25% of the data set falls.

### Level of catastrophic health expenditure for hypertension

Table 3 shows the level of CHE related to hypertension management and care, considering direct medical costs only and direct medical plus non-medical costs. Using the definition of poverty from Step 7, 65% of households were poor. Considering only direct health costs, 67% experienced catastrophic expenditures at a threshold of 10%. As the threshold increased to 40%, the percentage of households facing catastrophic expenditure declined to 58%. However, in Step 2, which incorporates both direct medical and non-medical costs, the percentages were slightly higher across all thresholds, with 68% experiencing catastrophic expenditure at a threshold of 10%.

**Table 3.** Catastrophic health expenditure related to hypertension management and care (N = 382).

Threshold	Direct medical costs	Direct non-medical costs
	(Percentage)	(Percentage)
Poor	65%	65%
Catastrophic 10	67%	68%
Catastrophic 20	63%	65%
Catastrophic 30	59%	62%
Catastrophic 40	58%	60%
Impoverished	95%	95%

Number of observations(N) in each method, standard deviation. Direct medical costs are catastrophic health expenditure due to direct health costs only. Direct non-medical costs are catastrophic health expenditure due to both direct medical health costs and direct non-medical costs, including transport.

### Factors associated with catastrophic health expenditure

As seen in Table 4, being unemployed is the only variable where the 95% confidence intervals (CIs) were not consistent with both an increase and a decrease in the risk of CHE (Direct medical costs only: OR = 0.30 [95% CI: 0.11–0.82]; Total health expenditure: OR = 0.26 [95% CI: 0.08–0.90]). Female sex was associated with a decreased risk of CHE, but the 95% CIs were also consistent with female sex being associated with a slightly increased risk of CHE (Direct medical costs only: OR = 0.37 [95% CI: 0.09–1.40]; Total health expenditure: OR = 0.18 [95% CI: 0.21–1.62]). Regarding marital status, results for the widowed category were not a significant predictor of a reduced risk of CHE, especially for total health expenditures (Direct medical costs only: OR = 0.52 [95% CI: 0.19–1.44]; Total health expenditure: OR = 0.32 [95% CI: 0.09–1.10]). Still, the 95% CIs are also consistent with an increased risk. The rest of the variables did not seem to be associated with an increase or decrease in the CHE risk. The pseudo R^2^ values were 0.0998 for direct medical costs only and 0.1714 for total health expenditures. The number of observations included in the analyses was 332.

**Table 4.** Determinants associated with catastrophic health expenditure for hypertension management and care in Greater Accra Region.

	Direct medical costs only (Threshold = 10%)			Total health expenditure (Threshold = 10%)	
Covariates	OR	(95% CI) P-value	OR		(95% CI) P-value
***Age***	0.98	(0.94–1.01) 0.20	0.98		(0.93–1.02) 0.32
***Gender***					
Male	1	Reference	1		Reference
Female	0.37	(0.09–1.40) 0.14	0.18		(0.21–1.62) 0.12
***Household size***	1.20	(0.93–1.54) 0.15	1.32*		(0.95–1.84) **0.09**
***Marital status***					
Married	1	Reference	1		Reference
Living together	1.03	(0.11–9.63) 0.97	0.61		(0.55–6.89) 0.69
Divorced	2.03	(0.42–9.74) 0.37	2.04		(0.23–18.26) 0.52
Widowed	0.52	(0.19–1.44) 0.21	0.32*		(0.09- 1.10) **0.07**
Never married/Single	1.01	(0.11–8.99) 0.92	0.52		(0.52- 5.22) 0.58
***Education***					
Primary	1	Reference	1		Reference
Secondary	0.81	(0.29–2.21) 0.67	0.70		(0.20–2.39) 0.57
Tertiary	0.94	(0.19–4.46) 0.93	1.54		(0.14–16.15) 0.71
***Employment***					
Employed	1	Reference	1		Reference
Unemployed	0.30*	(0.11–0.82) **0.01**	0.26*		(0.08–0.90) **0.03**
***Residence***					
Ga East Municipal Assembly	1	Reference	1		Reference
Ga South Municipal Assembly	1.31	(0.51–3.29) 0.57	1.89		(0.56–6.39) 0.30
Other	1.33	(0.34–5.14) 0.67	0.96		(0.22–4.08) 0.95
***Alcohol consumption***					
Yes	1	Reference	1		Reference
No	1.59	(0.46–5.44) 0.45	2.34		0.54–10.11 0.25
**Physical activity**					
Yes	1	Reference	1		Reference
No	0.99	(0.41–2.37) 0.98	1.34		(0.44–4.07) 0.59

*p < 0.05, **p < 0.01, ***p < 0.001 95% CI: 95% confidence interval Total health expenditure includes direct non-medical cost Pseudo R^2^=0.0998 for direct medical costs only Pseudo R^2^=0.1714 for total health expenditure.

## Discussion

This study examined the economic burden and determinants of catastrophic health expenditure (CHE) among households affected by hypertension. Understanding the financial impact of chronic conditions such as hypertension is crucial, as they require long-term management and medication, often leading to sustained out-of-pocket spending. Specifically, this study provides evidence of the economic burden of hypertension care among patients accessing hypertension clinics at primary healthcare centres in the Greater Accra Region. Our findings reveal that despite the implementation of social protection policies in Ghana, there is a high level of CHE among hypertensive patients in our study area. This case has shown that most hypertensive patients have a negative capacity to pay, which means that patients in such households could be forced to borrow and sell assets to cover medical expenses and are likely to fall into deeper poverty. Such households are likely to fall into a debt and economic hardship cycle, thereby making it impossible for them to escape poverty.

Our study reveals the high rate of unemployment among patients with hypertension in the Greater Accra Region. This may reflect broader developmental challenges in Ghana, including slow economic development and limited job creation in the formal sector. However, the observed association between unemployment and sex should be interpreted cautiously. In the Ghanaian context, many women, including stay-at-home mothers, widows, and older female caregivers, engage in household-based work that is not captured in formal labour statistics. As a result, they may be classified as unemployed despite contributing to household welfare, which may partly explain the higher proportion of women recorded as unemployed in the study.

More females than males were represented in our study, which could reflect differences in care-seeking behaviour, where females are generally more likely to contact health facilities [9]. It is important to note that the prominence of females (76.7%) could influence the interpretation and generalizability of the results. The observed high female prevalence aligns with previous studies, which documented that about 60% of women are affected by hypertension compared to men, due to biological risk factors [12]. This similarity suggests that the high female prevalence observed in our sample is reasonable and consistent with population trends. Our study shows that a large percentage of hypertensives are impoverished. The results align with the evidence of CHE among Ghanaian households, particularly highlighting disparities between the poorest and richest households, as shown in the GLSS [25,26]. The high average monthly household expenditure, combined with substantial direct medical and non-medical costs per hospital visit, reflects the considerable economic strain on families. The level of CHE estimated in this study appears to be high, which highlights the urgent need for targeted interventions to mitigate financial hardships and improve access to healthcare services. The findings indicate that both direct health costs and additional non-direct medical expenses contribute significantly to the financial burden faced by households, with implications for public health policy and resource allocation. Our study confirms that transportation costs are a major contributor to higher levels of CHE among patients with hypertension in the GAR. Existing studies have shown that transport costs are a key contributor to health expenditure in Ghana [23].

Our findings reveal that household size, marital status, and employment are associated with CHE. Although we did not categorize household size in our analysis, our findings suggest that larger households may incur higher spending, thereby increasing the likelihood of CHE. This finding is consistent with existing evidence indicating that the number of household members is a significant predictor of CHE in Africa [39]. We found that widowhood reduces the odds of incurring CHE. Again, the difference in the CHE risk is greater between the divorced and the widowed segments of the population, which might reflect the burden of losing household providers or breadwinners. Our findings further indicate that unemployment decreases the odds of CHE. It seems possible that this is because unemployed patients often have limited financial resources, which can lead to reduced access to healthcare services. If they cannot afford to seek care or buy medications, their out-of-pocket expenses for healthcare may be lower, even though this results in worse health outcomes.

## Strengths and limitations

A key strength of this study is that the primary data were collected using methods that ensured internal validity. However, this study has several limitations. First, although we restricted the study participants to estimate costs within six months of utilizing the hypertension clinics, the study could be affected by recall bias. Second, there may be a potential selection bias arising from the sampling approach and participation patterns – that is, those individuals who were more accessible and willing to participate may differ from those who were not included. Third, we did not consider the cost of care incurred in private health facilities. We also restricted our assessment to primary healthcare facilities in the Greater Accra Region. Therefore, our study may not be representative of the national population with hypertension. Fourth, the analytical steps adopted in our estimations have received extensive criticism for not considering personal savings or support provided by family and friends. Despite these limitations, our study presents potentially impactful evidence on CHE that may be useful for health policy decision-making in Ghana.

## Conclusion

This study highlights the substantial financial burden associated with hypertension care in the Greater Accra Region. The findings of this study contribute to the body of knowledge on the level and determinants of CHE for hypertension in Ghana. The study shows that direct medical and direct non-medical costs, including transportation expenditures, contribute to CHE for hypertension management; therefore, targeted interventions are required to address the financial burden of seeking care among those affected. The study recommends that the government should implement sustainable financing mechanisms to address high expenditure among individuals with hypertension. More research, particularly qualitative research, is needed to further the understanding of the impoverishing effects of health expenditure in Ghana.

## Supplementary Material

Supplementary file STROBE Checklist.docx

Survey tool.docx

## Data Availability

The dataset is available upon request.
