# Supplementary material for: Estimating the level and determinants of catastrophic expenditure related to hypertension management in the Greater Accra Region of Ghana
Source: Glob Health Action. 2025 Dec 18;18(1):2602116. doi: 10.1080/16549716.2025.2602116 (PMC12716478; doi:10.1080/16549716.2025.2602116)
Supplement: Supplementary file STROBE Checklist.docx [file ZGHA_A_2602116_SM0322.docx]

| **Supplementary file 1**-STROBE statement-Checklist items that should be included in reports of cross-sectional studies | | | | | |
| --- | --- | --- | --- | --- | --- |
|  | **Item**  **No.** | **Recommendation** | **Page No.** | **Relevant text from manuscript** |  |
| **Title and abstract** | 1 | *(a)* Indicate the study’s design with a commonly used term in the title or the abstract | 2 | Abstract |  |
|  |  | *(b)*Provide in the abstract with an informative and balanced summary of what was done and what was found | 2 | Abstract |  |
| **Introduction** |  |  |  |  |  |
| Background/rationale | 2 | Explain the scientific background and rationale for the investigation being reported | 3, 4 | Background: Paragraph 1 |  |
| Objectives | 3 | State specific objectives, including any prespecified hypotheses | 4 | Background: Paragraph 1 |  |
| **Methods** |  |  |  |  |  |
| Study design | 4 | Present key elements of study design early in the paper | 4 | Methods section |  |
| Setting | 5 | Describe the setting, locations, and relevant dates, including periods of recruitment, exposure, follow-up, and data collection | 4 | Metho section |  |
| Participants | 6 | (*a*) Give the eligibility criteria, and the sources and methods of selection of participants | 4 | Method section |  |
| Variables | 7 | Clearly define all outcomes, exposures, predictors, potential confounders, and effect modifiers. Give diagnostic criteria, if applicable. | 5, 6 | Method section |  |
| Data sources/measurement | 8* | For each variable of interest, give sources of data and details of methods of assessment (measurement). Describe the comparability of assessment methods if there is more than one group. | 5,6 | Method section |  |
| Bias | 9 | Describe any efforts to address potential sources of bias | 5,6 | Method section |  |
| Study size | 10 | Explain how the study size was arrived at | 5 | Method section |  |
| Quantitative variables | 11 | Explain how quantitative variables were handled in the analyses. If applicable, describe which groupings were chosen and why | 6,7 | Method section |  |
| Statistical methods | 12 | (*a*) Describe all statistical methods, including those used to control for confounding | 5,6,7 | Method section |  |
|  |  | (*b*) Describe any methods used to examine subgroups and interactions | 5,6,7 | Method section |  |
|  |  | (*c*) Explain how missing data were addressed | 5,6,7 | Method section |  |
|  |  | (*d*) If applicable, describe analytical methods taking account of sampling strategy | 5,6,7 | Method section |  |
|  |  | (*e*) Describe any sensitivity analyses | NA | Not applicable |  |
| **Results** |  |  |  |  |  |
| Participants | 13* | (a) Report numbers of individuals at each stage of study—eg numbers potentially eligible, examined for eligibility, confirmed eligible, included in the study, completing follow-up, and analysed | 7 | Results section |  |
|  |  | (b) Give reasons for non-participation at each stage | NA | Not applicable |  |
|  |  | (c) Consider use of a flow diagram | NA | Not applicable |  |
| Descriptive data | 14* | (a) Give characteristics of study participants (eg demographic, clinical, social) and information on exposures and potential confounders | 7 | Results section:  Table 1 |  |
|  |  | (b) Indicate number of participants with missing data for each variable of interest | NA | Not applicable |  |
| Outcome data | 15* | Report numbers of outcome events or summary measures | 7,8 | Results section: Table 1, Table 2, Table 3 |  |
| Main results | 16 | (*a*) Give unadjusted estimates and, if applicable, confounder-adjusted estimates and their precision (eg, 95% confidence interval). Make clear which confounders were adjusted for and why they were included | 8 | Results section: Table 4 |  |
|  |  | (*b*) Report category boundaries when continuous variables were categorized | NA | Not applicable |  |
|  |  | (*c*) If relevant, consider translating estimates of relative risk into absolute risk for a meaningful time period | NA | Not applicable |  |
| Other analyses | 17 | Report other analyses done—eg analyses of subgroups and interactions, and sensitivity analyses | NA | Not applicable |  |
| **Discusssion** |  |  |  |  |  |
| Key results | 18 | Summarise key results with reference to study objectives | 9 | Discussion section |  |
| Limitations | 19 | Discuss limitations of the study, taking into account sources of potential bias or imprecision. Discuss both direction and magnitude of any potential bias | 9,10 | Discussion section |  |
| Interpretation | 20 | Give a cautious overall interpretation of results considering objectives, limitations, multiplicity of analyses, results from similar studies, and other relevant evidence | 9,10 | Discussion section |  |
| Generalisability | 21 | Discuss the generalisability (external validity) of the study results | 9,10 | Discussion section |  |
| **Other information** |  |  |  | Funding section |  |
| Funding | 22 | Give the source of funding and the role of the funders for the present study and, if applicable, for the original study on which the present article is based | 11 |  |  |

*Give information separately for exposed and unexposed groups

Note: An Explanation and Elaboration article discusses each checklist item and gives methodological background and published examples

of transparent reporting. The STROBE checklist is used in conjunction with this article (freely available on the Web sites of PLoS Medicine

at <http://www.plosmedicine.org/>, Annals of Internal Medicine at <http://www.annalys.org>, and Epidemiology at<http://www.epidem.com/>).

Information on the STROBE Initiative is available at [www.strobe-statement.org](http://www.strobe-statement.org).
