# Supplementary material for: Estimating the level and determinants of catastrophic expenditure related to hypertension management in the Greater Accra Region of Ghana
Source: Glob Health Action. 2025 Dec 18;18(1):2602116. doi: 10.1080/16549716.2025.2602116 (PMC12716478; doi:10.1080/16549716.2025.2602116)
Supplement: Survey tool.docx [file ZGHA_A_2602116_SM0321.docx]

**UNIVERSITY OF BERGEN**

**DEPARTMENT OF GLOBAL HEALTH AND PRIMARY CARE**

**BERGEN CENTRE FOR ETHICS AND PRIORITY SETTING (BCEPS)**

**CATASTROPHIC HEALTH EXPENDITURE SURVEY FOR HYPERTENSION SERVICES IN GHANA**

| **Preamble**  Participating in this study is entirely voluntary, and your confidentiality will be strictly maintained. The questions asked to reveal your demographic profile and the direct medical and direct non-medical expenditures you incur for hypertension care in Ghana.  The majority of the questions are relatively easy and straight to the point. Please answer all questions honestly. This survey is to be administered to hypertensives undergoing management with antihypertensives who are aged above 45 years and utilize one of the following hospitals: Ga East Municipal Hospital (GEMH) and Weija-Gbawe Municipal Hospital (WGMH) in the Ga East Municipal Assembly and Ga South District Assembly. |
| --- |

**Details of the instrument**

1. Date:

2. Questionnaire number:

3. Interviewer:

4. Health facility name:

| **SECTION A- SOCIO-DEMOGRAPHIC/ECONOMIC INFORMATION** | | |
| --- | --- | --- |
| **S/N** | **Questions and items** | **Coding categories** |
| 1. | Gender | Male____________1  Female__________ 2 |
| 2. | Age | _____________(years) |
| 3. | Please indicate your marital status | Married _________________1  Living together ___________ 2  Divorced or separated______3  Widowed________________ 4  Never married/never lived together ___5 |
| 4. | Your highest level of education | Primary__________________1  Middle___________________2  JSS/JHS___________________3  SECONDARY ______________4  SSS/SHS__________________5  HIGHER __________________6 |
| 5. | Indicate your residence | Ga East Municipal Assembly_1  Ga South Municipal Assembly_2  Others____________________3 |
| 6. | State your employment status. |  |
| 7. | Indicate your ethnic status | Akan_____________________1  Ga/dangme_______________­_2  Ewe______________________3  Guan_____________________4  Mole-Dagbani_____________5  Grussi____________________6  Gruma___________________7  Mande___________________8  Other____________________99 |
| 8. | What is your religion? | Catholic______________1  Anglican______________2  Methodist ____________3  Presbyterian___________4  Pentecostal/Charismatic___5  Other Christian__________6  Islam__________________7  Traditional/Spiritualist____8  No Religion ____________9  Other_________________99 |
| **SECTION B- BEHAVIOURAL FACTORS** | | |
| 9. | Do you smoke? | Yes__________________1  No__________________2 |
| 10. | Do you drink alcohol? | Yes_________________1  No__________________2 |
| 11. | Please indicate if you undertake any physical activity | Yes__________________1  No___________________2 |
| 12. | Any views on the diets that you eat | Fruits, vegetables, and low-fat dairy foods________________1  Saturated fat, cholesterol, and tans fats_________________2  Whole-grain foods, fish, poultry, and nuts_________________3  Sodium, sweets, sugary drinks, and red meats____________4 |
|  | **SECTION C- HEALTH EXPENDITURE SURVEY** | |
| 13. | How much did you pay for your current visit? |  |
|  | (a)consultation |  |
|  | (b)laboratory test |  |
|  | (c)medicines |  |
|  | (d)transport |  |
|  | (e)food cost |  |
|  | (f)other health care costs |  |
| 14. | How many times do you visit the facility due to hypertension? |  |
| 15. | Can you tell me how much time it took you to undertake the following activities: (In minutes) |  |
|  | (a)Travelling |  |
|  | (b)Waiting |  |
|  | (c)During consultation |  |
|  | How much time do you take away from your work (Self, formal, or informal employment) to come for your visit (Hours and minutes)? |  |
| 16. | Who pays for your hypertension management? | 1. Respondent 2. Spouse/partners 3. Parents 4. Son/Daughter 5. Brother/sister 6. Employer 7. Health insurance 8. Free 9. Specify (Other) |
| 17. | You may share any information regarding expenses incurred for hypertension management at the facility. |  |
